# Prognostic factors and long‐term outcomes with endoscopic submucosal dissection for colorectal tumors in patients aged 75 years or older

**DOI:** 10.1002/deo2.70137

**Published:** 2025-05-06

**Authors:** Takaya Miura, Satohiro Matsumoto, Azumi Sato, Shu Kojima, Goya Sasaki, Mina Morino, Keita Matsumoto, Hitomi Kashima, Yudai Koito, Takehiro Ishii, Shuhei Yoshikawa, Haruka Otake, Takeshi Uehara, Masanari Sekine, Takeharu Asano, Hiroyuki Miyatani, Hirosato Mashima

**Affiliations:** ^1^ Department of Gastroenterology Jichi Medical University Saitama Medical Center Saitama Japan

**Keywords:** colorectal tumors, endoscopic submucosal dissection, long‐term outcomes, older patients, prognostic factors

## Abstract

**Background:**

Studies regarding the long‐term outcomes of endoscopic submucosal dissection (ESD) performed in older patients with colorectal tumors are limited. Therefore, in this study, we aimed to analyze the long‐term outcomes of older patients with colorectal tumors who underwent ESD and identify prognostic factors.

**Methods:**

The data of patients aged ≥ 75 years who underwent ESD for colorectal tumors (adenoma and Tis/T1 colorectal cancer) at a single center were retrospectively analyzed. Prognostic factors for overall survival were analyzed using the Kaplan–Meier method and the Cox proportional hazard model.

**Results:**

Of the 156 patients included, 51 patients died during the follow‐up period, among whom two deaths were due to colorectal cancer. The univariate analysis revealed that an age ≥83 years, Charlson Comorbidity Index ≥2, prognostic nutritional index <46, and neutrophil‐to‐lymphocyte ratio (NLR) ≥3 were associated with poor overall survival. The multivariate analysis identified Charlson Comorbidity Index ≥2 (hazard ratio: 2.26; 95% confidence interval (CI): 1.24–4.13; *p *= 0.0008) and NLR ≥3 (hazard ratio, 1.98; 95% CI: 1.02–3.81; *p *= 0.042) as independent prognostic factors.

**Conclusions:**

CCI and NLR may be useful parameters for decision‐making in older patients undergoing colorectal ESD.

## INTRODUCTION

The global older population is growing,^1^ which impacts daily clinical practice. As of 2023, 29.1% of the Japanese population was older adults, including 16.1% who were ≥75 years of age.^2^ Japan has become a super‐aging country, as the proportion of older individuals increases annually. As a result, the prevalence of colorectal cancer (CRC) is increasing.^3^ The selection of therapeutic strategies for older patients with colorectal tumors (adenoma and Tis/T1 CRC) and comorbidities has become increasingly challenging.

Endoscopic submucosal dissection (ESD) for colorectal tumors is widely performed as a definitive and minimally invasive procedure. Although the short‐term and long‐term therapeutic outcomes of ESD have been reported to be favorable, even in older patients, the incidence of complications associated with ESD, such as bleeding and perforation, ranges from 3.7% to 9.8%.^4^, ^5^, ^6^ The mortality rate due to complications is assumed to be low, though the increased time in bed or length of hospital stay due to complications may impair an individual's activities of daily living. Given the high mortality in older adults, follow‐up without treatment may sometimes be acceptable. Although the Charlson Comorbidity Index (CCI),^7^ prognostic nutritional index (PNI),^8^ neutrophil‐to‐lymphocyte ratio (NLR),^9^ and geriatric nutritional risk index (GNRI)^10^ have been reported to be useful prognostic factors, there are no known indicators that are effective for accurately predicting the outcomes of colorectal ESD. Moreover, previous studies reported the short‐term clinical outcomes of ESD in older patients with colorectal tumors, and few studies have focused on the long‐term outcomes and prognostic factors of older patients with colorectal tumors.^11^, ^12^ Therefore, we conducted this study with the aim of analyzing the long‐term outcomes of older patients with colorectal tumors who underwent ESD to identify prognostic factors.

## MATERIALS AND METHODS

### Patients

This retrospective, single‐center study was conducted at Jichi Medical University Saitama Medical Center. ESD for colorectal tumors was performed as previously reported.^13^, ^14^

The data of consecutive patients aged ≥75 years with colorectal tumors who underwent ESD at Jichi Medical University Saitama Medical Center between December 2005 and June 2016 were retrospectively reviewed.

The medical records of the patients were reviewed to obtain data regarding the clinical and demographic characteristics, including age, sex, Eastern Cooperative Oncology Group Performance Status (ECOG‐PS),^15^ and follow‐up period. The CCI, PNI, NLR, and GNRI were evaluated as prognostic factors. The CCI was calculated based on its original definition.^7^ The PNI was calculated using the serum albumin level and total lymphocyte count (PNI = 10 × serum albumin (g/dL) + 0.005 × total lymphocyte count per mm^3^). The NLR was calculated by dividing the total neutrophil count by the total lymphocyte count. The GNRI was calculated using the serum albumin level and body mass index (GNRI = 14.89 × serum albumin (g/dL) + 41.7 × body mass index / 22).

An opt‐out document on the hospital website was used to obtain from all patients undergoing ESD. The protocol of this study was approved by the ethics committee of Jichi Medical University Saitama Medical Center (S24‐155).

### Clinicopathological characteristics and short‐term outcomes

Data regarding the patients’ clinical and demographic characteristics, including age, sex, height, weight, ECOG‐PS, CCI, PNI, NLR, and GNRI, were obtained from the medical records.

The lesion characteristics, such as location, size, macroscopic type, histologic type, invasion depth, and lymphatic or vascular involvement, were also obtained. The short‐term outcomes evaluated in this study included adverse events such as perforation and bleeding, curability, and whether additional surgery was performed.

### Curability criteria

The curability of ESD was determined according to the guidelines of the Japanese Society for Cancer of the Colon and Rectum.^16^ Resection was determined to be curative when the lesion had a negative vertical resection margin, well‐/moderately‐differentiated or papillary cancer, submucosal invasion depth <1000 µm, absence of lymphatic or vascular infiltration, and budding grade 1.^16^ When the resection was determined to be non‐curative, the indications for additional colectomy were determined according to the guidelines at the time of ESD and the patient's wishes.^17^, ^18^, ^19^ The patients were classified into three groups according to the degree of curability by ESD: curability A, defined as curative resection; curability B, defined as non‐curative resection with additional surgery; and curability C, defined as non‐curative resection without additional surgery.

### Follow‐up and long‐term outcomes

Patients with adenoma or Tis CRC who achieved curative resection by ESD underwent regular colonoscopy. Patients with T1 CRC regularly underwent computed tomography in addition to colonoscopy, regardless of whether a curative resection was achieved or not (with or without additional surgery). Colonoscopy should be conducted each year, and computed tomography at 6‐month intervals. However, the imaging was not performed at consistent intervals in the current study as the participants were older patients with various backgrounds and underlying diseases. Long‐term outcomes, including recurrence of CRC (local or metastatic) and survival status, were collected by reviewing the patients’ medical records, interviewing patients or their families over the phone, and mailing a questionnaire survey. These surveys were conducted from March 2021 to June 2021. Local recurrence of CRC was defined as cancer that recurred at a scar caused by ESD, and metastatic recurrence was defined as cancer located in lymph nodes or distant organs.

### Statistical analysis

Categorical variables are expressed as numbers and percentages while continuous variables are expressed as median and interquartile range.

Overall survival (OS) was defined as the time from the date of ESD to the date of death from all causes or the date of last known survival. For the OS analysis, all patients were followed for a minimum of 5 years or until death. Patients with a follow‐up duration of less than 5 years were included only if they had died during that period. The OS after ESD was analyzed using the Kaplan–Meier method, and the differences between the groups were evaluated using the log‐rank test. The association between OS and each clinicopathological factor was analyzed by univariate analysis using the log‐rank test. OS was also compared among the three curability groups using the log‐rank test. Then, multivariate analysis was carried out to identify the risk factors for OS. Variables that were statistically significant in the univariate analysis were included in the multivariate analysis to minimize overfitting due to the limited number of outcome events. When there were more than two groups to be analyzed, the level of significance was adjusted using Holm's method. When there were more than two groups to be analyzed, the cut‐off values for OS, age, sex, ECOG‐PS, CCI, PNI, NLR, and GNRI were determined using receiver operating characteristic analysis. The cut‐off values with the highest sensitivity and specificity for OS were used. Clinicopathological factors that showed significant differences in the univariate analysis were included in the multivariate analysis using a Cox proportional hazard regression model. Statistical significance was set at *p* < 0.05. All statistical analyses were performed using EZR (version 1.61; Jichi Medical University Saitama Medical Center, Saitama, Japan), a graphical user interface for R (version 2.13.0; The R Foundation for Statistical Computing).^20^

## RESULTS

**FIGURE 1:**
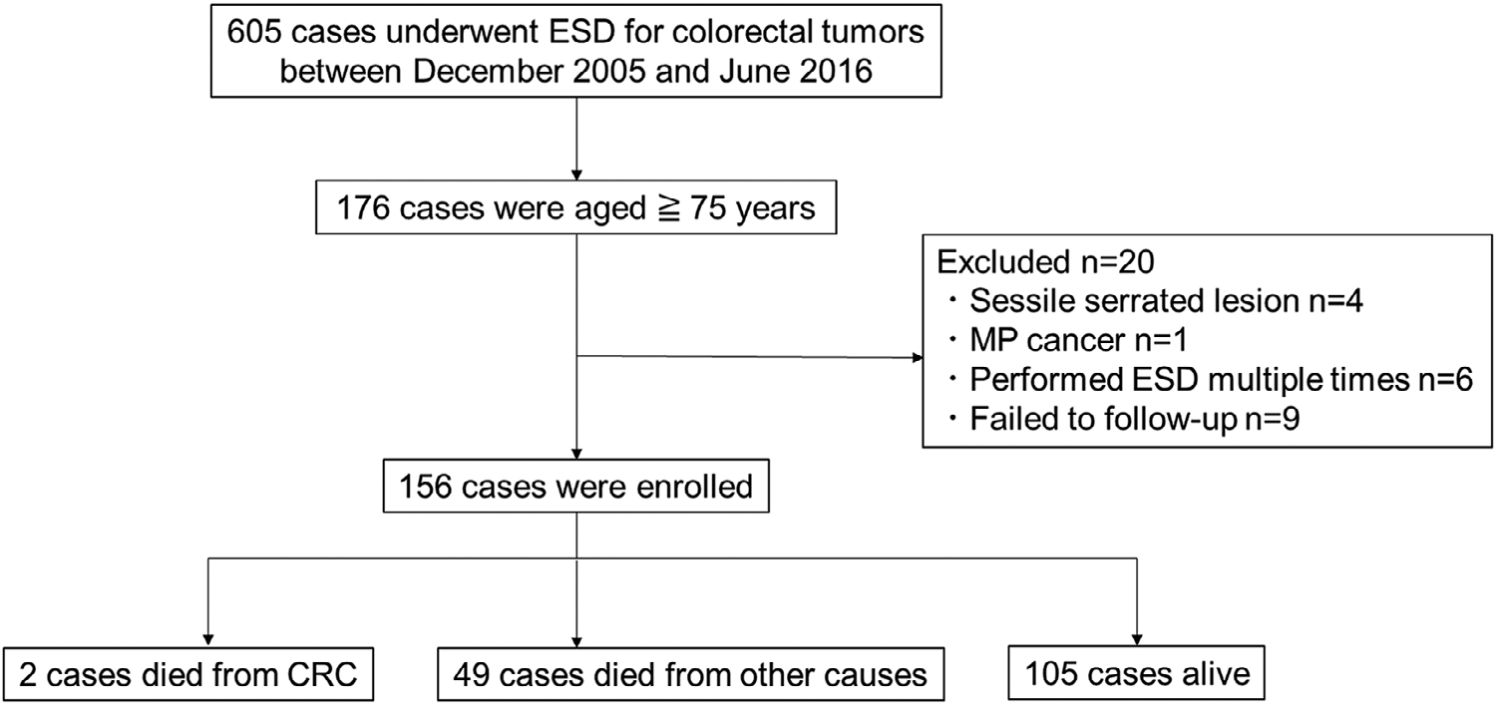
Flow of patients enrolled in the study. ESD, endoscopic submucosal dissection; CRC, colorectal cancer; MP, muscularis propria invasion.

Of 176 patients selected, 156 were ultimately included in the analysis. Four patients with sessile serrated lesions confirmed by pathological examination after ESD, one patient with advanced cancer (invasion depth: muscularis propria) confirmed by pathological examination after ESD, six patients who underwent repeated ESD, and nine patients who were lost to follow‐up were excluded from the analysis (Figure 1). Of the nine patients excluded due to loss to follow‐up, eight were in Group A and had no recurrence during the 5‐year follow‐up before transferring to other hospitals. One patient in Group C declined additional surgery and discontinued follow‐up after 1 year due to advanced age and difficulty visiting the hospital. No recurrence of colorectal cancer was observed during the follow‐up period.

The median patient age was 79 years (range: 75–89 years), and 57.7% were men (Table 1). The ECOG‐PS was 0 or 1 in 151 patients (96.8%). The median follow‐up period was 6.6 years (range: 0.1–14.4 years). The CCI was 0 or 1 in 118 patients (75.6%). The median PNI was 48.2 (range: 38.3–101.9), the median NLR was 2.37 (range: 0.28–8.39), and the median GNRI was 101.3 (range: 75.7–114.8). NLR data was not reported for seven patients.

**TABLE 1:** Demographic and clinical characteristics of 156 patients aged 75 years who underwent endoscopic submucosal dissection for early colorectal cancer.

	*n* = 156
Age, years, median (range)	79 (75–89)
Sex, *n* (%)	
Male	90 (57.7)
Female	66 (42.3)
ECOG PS, *n* (%)	
0–1	151 (96.8)
2 or above	5 (3.2)
CCI, *n* (%)	
0–1	118 (75.6)
2 or above	38 (24.4)
PNI, median (range)	48.2 (38.3–101.9)
NLR^†^, median (range)	2.37 (0.28–8.39)
GNRI, median (range)	101.3 (75.7–114.8)
Follow‐up period, years, median (range)	6.63 (0.08–14.4)

^†^ Seven patients with missing data for NLR were excluded.

Abbreviations: CCI, Charlson comorbidity index; CRC, colorectal cancer; ECOG PS, Eastern Cooperative Oncology Group performance status; ESD, endoscopic submucosal dissection; GNRI, geriatric nutritional risk index; NLR, neutrophil to lymphocyte ratio; PNI, prognostic nutritional index.

The majority of lesions developed in the colon (119 lesions, 75.8%), and the median tumor size was 25 mm (Table 2). All tumors were histologically proven as adenoma or differentiated carcinoma, and 27 lesions (17.3%) invaded the submucosa. Lymphatic or vascular infiltration was observed in 10 lesions (6.4%). Six lesions had a positive horizontal resection margin (3.8%) and one had a positive vertical resection margin (0.6%). Postoperative bleeding (four patients, 2.6%) and perforation (seven patients, 4.5%) were observed. Additional colectomy was performed in eight patients (5.1%), including three (1.9%) who had residual cancer and one (0.6%) who had lymph node metastasis (LNM). No patients developed local or distant metastatic recurrence during the follow‐up period. Of 51 patients (32.7%) who died during the follow‐up period, 49 patients (31.4%) died from causes other than CRC and two (1.3%) died from metachronous CRC. The causes of death included cancer in other organs (13 patients), pneumonia (eight patients), cardiovascular disease (seven patients), cerebrovascular disease (three patients), and others (18 patients). One patient who died from CRC was classified into group C in curability, as surveillance colonoscopy performed after ESD revealed advanced CRC at another site, and surgery was performed. Six years later, this patient developed a recurrence of liver metastasis and died. The other patient who died from CRC was classified into group C and found to have metachronous advanced CRC at the site of ESD and at another site. The patient died 11 years after ESD, but the time of recurrence was unknown.

**TABLE 2:** Clinicopathological characteristics of 156 patients aged 75 years who underwent endoscopic submucosal dissection for early colorectal cancer.

	*n* = 156
Tumor location, *n* (%)	
Colon	118 (75.8)
Rectum	38 (24.2)
Tumor size, mm, median (range, IQR)	25 (8–80, 15)
Macroscopic type, *n* (%)	
Polypoid	125 (80.1)
Non‐polypoid	31 (19.9)
Histology, *n* (%)	
adenoma	52 (33.3)
tub/pap	104 (66.7)
Depth of invasion, *n* (%)	
Adenoma/Tis	129 (82.7)
T1	27 (17.3)
Lymphovascular invasion, *n* (%)	10 (6.4)
Horizontal margin positive, *n* (%)	6 (3.8)
Vertical margin positive, *n* (%)	1 (0.6)
Curability (Group/A/B/C), *n* (%)	139 (89.1)/8 (5.1)/9 (5.8)
Adverse events	
Postoperative bleeding, *n* (%)	4 (2.6)
Perforation, *n* (%)	7 (4.5)
Additional surgery, *n* (%)	8 (5.1)
Residual lesion, *n* (%)	3 (1.9)
Lymph node metastasis, *n* (%)	1 (0.6)
Prognosis	
Death due to colorectal cancer, *n* (%)	2 (1.3)
Death due to any causes, *n* (%)	49 (31.4)

Abbreviations: Curability, Group A: curative resection, Group B: non‐curative resection with additional surgery, Group C: non‐curative resection without additional surgery; IQR, inter‐quartile range; pap, papillary adenocarcinoma; Tis, limited to mucosa; tub, tubular differentiated adenocarcinoma; T1, submucosal invasion.

**FIGURE 2:**
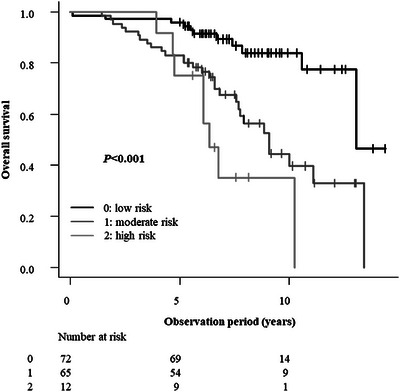
Overall survival curves for patients grouped by neutrophil‐to‐lymphocyte ratio and Charlson Comorbidity Index score. Seven patients with missing data for NLR were excluded.

OS was significantly poorer in patients aged ≥83 years and in those with a CCI ≥2, PNI <46, or NLR ≥3. In contrast, sex and ECOG‐PS showed no statistically significant association with OS in the univariate analysis. Furthermore, no statistically significant difference in OS was observed among the three curability groups (Groups A, B, and C; Table 3). Subsequently, a multivariate Cox proportional hazard analysis was performed using the factors found to be significant in the univariate analysis (age, CCI, PNI, and NLR). CCI ≥2 (hazard ratio: 2.26; 95% confidence interval [CI]: 1.24–4.13; *p *= 0.0008) and NLR ≥3 (hazard ratio: 1.98; 95% CI: 1.02–3.81; *p *= 0.042) were identified as independent prognostic factors for poorer OS (Table 4). Based on these results, patients were categorized into three risk groups using a simple scoring system: score 0 (low risk), defined as CCI <2 and NLR <3; score 1 (moderate risk), defined as CCI ≥ 2 or NLR ≥3; and score 2 (high risk), defined as CCI ≥2 and NLR ≥3. Age and PNI were not included in the scoring system because they were not determined to be significant in the multivariate analysis. Significant differences in survival outcomes were observed among the low‐, moderate‐, and high‐risk groups (all *p *< 0.001). Trend analysis further indicated poorer outcomes for patients with higher risk. Multiple comparisons using Holm's method identified differences among the three groups. OS tended to be poorer with increased risk of mortality based on these factors (*p *= 0.0000031), based on the log‐rank test, and was significantly poorer in both the moderate‐risk and high‐risk groups than in the low‐risk group (*p *= 0.00037 and 0.000012, respectively) when Holm's method was used. There was no significant difference in OS between the moderate‐risk and high‐risk groups (*p *= 0.11; Figure 2).

**TABLE 3:** Overall survival (Kaplan–Meier method).

Variable	No. of patients	5‐year OS	*p*‐value
Age			0.00005
<83 years	125	0.90	
≥83 years	31	0.84	
Sex			0.057
Male	90	0.86	
Female	66	0.94	
ECOG PS			0.455
0–1	151	0.89	
≥2	5	0.8	
CCI			0.00084
0–1	118	0.91	
≥2	38	0.84	
PNI			0.00068
≥46	115	0.91	
<46	41	0.83	
NLR			0.00052
≥3	55	0.8	
<3	94	0.94	
GNRI			0.163
≥96	137	0.90	
<96	19	0.84	
Curability			0.53
Group A	139	0.89	
Group B	8	0.88	
Group C	9	1	

Abbreviations: CCI, Charlson Comorbidity Index; Curability, Group A: curative resection, Group B: non‐curative resection with additional surgery, Group C: non‐curative resection without additional surgery; ECOG PS, Eastern Cooperative Oncology Group performance status; GNRI, geriatric nutritional risk index; NLR, neutrophil to lymphocyte ratio; OS, overall survival; PNI, prognostic nutritional index.

**TABLE 4:** Multivariate analysis of factors associated with overall survival.

Variable	HR	95% CI	*p‐*value
Age ≥83 years	1.71	0.91–3.23	0.096
CCI ≥2	2.26	1.24–4.13	0.0008
PNI ≤46	1.46	0.75–2.83	0.268
NLR ≥3	1.98	1.02–3.81	0.042

Abbreviations: CCI, Charlson Comorbidity Index; CI, confidence interval; HR, hazard ratio; NLR, neutrophil‐to‐lymphocyte ratio; PNI, prognostic nutritional index.

## DISCUSSION

This study identified both the CCI and NLR as independent prognostic factors for overall survival in older patients undergoing colorectal ESD. While a recent study by Asayama et al. also identified CCI as a significant prognostic factor in a similar patient cohort,^21^ our study additionally evaluated multiple nutritional and inflammatory markers, identifying NLR as another independent predictor. This suggests that assessing both comorbidity burden (CCI) and systemic inflammation (NLR) may allow for a more multifaceted risk evaluation. Based on these findings, we developed a novel risk classification combining CCI and NLR, which may assist in predicting long‐term outcomes after ESD. This combined score offers a potentially enhanced approach to risk stratification compared to using CCI alone.

CCI is a scoring system used to classify comorbidities that may affect mortality risk.^7^ Several reports have indicated that CCI is a useful prognostic factor for gastrointestinal cancer.^22^, ^23^, ^24^, ^25^ Our finding that CCI is closely associated with OS in older patients undergoing colorectal ESD aligns with the conclusions of a recent similar study by Asayama et al.^21^ This consistency underscores the importance of assessing the comorbidity burden in this patient population.^11^ In the current study, 96% of older patients who died after undergoing colorectal ESD died from causes other than CRC. CCI, which allows for the evaluation of the risk of various comorbidities, is closely associated with OS in this study.

NLR is a simple indicator of systemic inflammatory response, including trauma and sepsis.^26^, ^27^ NLR is also a useful prognostic factor for gastrointestinal cancer.^9^, ^28^, ^29^ As studies regarding the usefulness of NLR for determining the long‐term prognosis of patients with CRC mostly involved patients with advanced cancer,^30^, ^31^, ^32^ it is unclear if NLR is useful for the evaluation of the prognosis of early CRC. However, NLR has been reported to be a useful prognostic factor not only for cancer but also for arteriosclerotic diseases such as heart disease and cerebrovascular diseases.^33^, ^34^, ^35^ Although the current study included patients with early CRC, several patients died from heart disease or cerebrovascular diseases. NLR appears to be useful for evaluating the potential mortality risk.

The use of GNRI and PNI as indicators was also evaluated in this study. PNI was identified as a prognostic factor by univariate analysis but was not identified as an independent prognostic factor via the multivariate analysis. Moreover, although a previous study reported that GNRI was the most important prognostic factor in patients who underwent colorectal ESD,^11^ no association between GNRI and the survival rate after colorectal ESD was observed in this study. CCI and NLR were identified as prognostic factors in the current study. These factors allow for the evaluation of patient characteristics, diseases, and inflammatory markers using multifaceted perspectives.

As the number of patients who underwent non‐curative ESD was small in this study, the necessity of additional surgery could not be evaluated. However, of the eight patients who underwent additional surgery, only one had lymph node metastasis; therefore, additional surgery may not be necessary and the decision requires careful consideration, especially in patients with a high CCI or NLR predicting limited long‐term survival. Recent reports regarding gastric cancer have presented models predicting LNM after non‐curative ESD, which have been reported to be useful for selecting patients who require additional surgery.^36^, ^37^ For CRC, tools such as the nomogram developed by Kajiwara et al.^38^ are emerging as valuable aids for estimating LNM risk and facilitating shared decision‐making regarding additional surgery. Furthermore, it may be necessary to clarify the stratification of survival rates based on the evaluation of the risk of metastasis and the prognostic factors identified in the current study in future studies. This integrated approach could be particularly relevant for older patients with low‐risk lesions such as colorectal adenoma and intramucosal carcinoma, which typically develop at very slow rates.^39^ Therefore, the prognostic information derived from CCI and NLR in our study may contribute to informed discussions about management options, including the potential benefits and burdens of ESD versus surveillance, for older patients with these low‐risk lesions, considering their overall life expectancy. However, all patients with CRC‐related death in this study died from metachronous, recurrent CRC that was not associated with the lesions treated with ESD. Therefore, therapeutic strategies must be developed to detect the development of CRC after ESD.

This study has several limitations. First, the retrospective study design may have introduced a selection bias and limited generalizability. Prospective studies are needed to validate the usefulness of CCI and NLR as prognostic factors in older patients with colorectal tumors undergoing ESD. Second, this was a single‐center study, limiting the sample size and patient characteristics. Larger multicenter studies are needed to confirm the results of this study. Third, our multivariate analysis included only variables significant in the univariate analysis (age, CCI, PNI, and NLR) due to the limited number of outcome events. Fourth, the lack of a non‐ESD comparison group prevents conclusions about the indication for ESD itself versus other management strategies in patients with high CCI/NLR scores.

## CONCLUSION

This study demonstrated that high CCI and NLR are independent prognostic factors associated with decreased OS in older patients aged ≥ 75 years with colorectal tumors who undergo ESD. These two prognostic factors may provide valuable insights to guide shared decision‐making regarding management strategies for older patients treated with ESD.

## CONFLICT OF INTEREST STATEMENT

None.

## ETHICS STATEMENT

Approval of the research protocol by an Institutional Reviewer Board: This study was approved by the Ethics Committee of Jichi Medical University Saitama Medical Center (Approval No: S24‐155).

## PATIENT CONSENT STATEMENT

Informed consent was obtained from all participants through an opt‐out system on the hospital website.

## CLINICAL TRIAL REGISTRATION

N/A.
